# EDUCATION, LITERACY, NUMERACY AND HEALTH INFORMATION SEEKING IN LATER LIFE

**DOI:** 10.1093/geroni/igz038.1174

**Published:** 2019-11-08

**Authors:** Takashi Yamashita, Anthony R Bardo, Roberto J Millar, Shalini Sahoo, Phyllis Cummins, Darren Liu

**Affiliations:** 1 University of Maryland, Baltimore County, Baltimore, Maryland, United States; 2 University of Kentucky, Lexington, Kentucky, United States; 3 University of Maryland, Baltimore, Baltimore, Maryland, United States; 4 Scripps Gerontology Center, Miami University, Oxford, Ohio, United States; 5 Des Moines University, Des Moines, Iowa, United States

## Abstract

Health information plays a critical role for health promotion and maintenance in later life. While health information seeking is primarily driven by need (e.g., health), significantly less is known about the roles of education and health-literacy. Thus, we examine complex pathways that link health information seeking behaviors with education and health literacy (decomposed into general literacy and numeracy), and how these pathways differ by health status among a nationally representative sample of Americans age 50 and older (n = 2,750). Data come from the 2012/2014 Program for International Assessment of Adult Competencies. Multi-group structural equation models were used to examine the use of eight health information sources (newspapers, magazines, internet, radio, TV, books, friends/family, and health professionals) by health status (good vs. poor). Findings showed that literacy and numeracy are significant mediators of the relationship between education and health professional as an information source. Additionally, the mediation effects on health professionals by literacy status [indirect-effect (good vs. poor health) = 0.48 vs. 2.13, p < 0.05] and numeracy [indirect-effect (good vs. poor health) = -0.47 vs. -1.81, p < 0.05] were significantly moderated by health. At the same time, no moderated mediation effect was observed in the use of any other information sources. This study provides some of the first nationally representative evidence regarding how education functions through health literacy components to shape health information seeking behaviors by health status. Explanations and implications for differing effects of education, literacy, and numeracy on health information seeking in later life were evaluated.

